# Modulation of ACE-2 mRNA by inflammatory cytokines in human thyroid cells: a pilot study

**DOI:** 10.1007/s12020-021-02807-w

**Published:** 2021-07-05

**Authors:** Francesca Coperchini, Gianluca Ricci, Laura Croce, Marco Denegri, Rubina Ruggiero, Laura Villani, Flavia Magri, Luca Chiovato, Mario Rotondi

**Affiliations:** 1 Laboratory for Endocrine Disruptors, Unit of Internal Medicine and Endocrinology, Istituti Clinici Scientifici Maugeri IRCCS, 27100 Pavia, PV Italy; 2 Department of Internal Medicine and Therapeutics, University of Pavia, 27100 Pavia, PV Italy; 3 Unit of Molecular Cardiology, Istituti Clinici Scientifici Maugeri IRCCS, 27100 Pavia, Italy; 4 Department of General and Minimally Invasive Surgery, Istituti Clinici Scientifici Maugeri IRCCS, 27100 Pavia, PV Italy; 5 Unit of Pathology, Istituti Clinici Scientifici Maugeri IRCCS, 27100 Pavia, PV Italy

**Keywords:** Thyroid, Thyrocytes, SARS-COV-2, COVID-19, ACE-2

## Abstract

**Introduction:**

Angiotensin-converting-enzyme-2 (ACE-2) was demonstrated to be the receptor for cellular entry of SARS-CoV-2. ACE-2 mRNA was identified in several human tissues and recently also in thyroid cells in vitro.

**Purpose:**

Aim of the present study was to investigate the effect of pro-inflammatory cytokines on the ACE-2 mRNA levels in human thyroid cells in primary cultures.

**Methods:**

Primary thyroid cell cultures were treated with IFN-γ and TNF-α alone or in combination for 24 h. ACE-2 mRNA levels were measured by RT-PCR. As a control, the levels of IFN-γ inducible chemokine (CXCL10) were measured in the respective cell culture supernatants.

**Results:**

The mean levels of ACE-2 mRNA increased after treatment with IFN-γ and TNF-α in all the thyroid cell preparations, while the combination treatment did not consistently synergically increase ACE-2-mRNA. At difference, CXCL10 was consistently increased by IFN-γ and synergically further increased by the combination treatment with IFN-γ + TNF-α, with respect to IFN-γ alone.

**Conclusions:**

The results of the present study show that IFN-γ and, to a lesser extent TNF-α consistently increase ACE-2 mRNA levels in NHT primary cultures. More interestingly, the combined stimulation (proven to be effective according to the synergic effect registered for CXCL10) produces different responses in terms of ACE-2 mRNA modulation. These results would suggest that elevated levels of pro-inflammatory cytokines could facilitate the entering of the virus in cells by further increasing ACE-2 expression and/or account for the different degree of severity of SARS-COV-2 infection. This hypothesis deserves to be confirmed by further specific studies.

## Introduction

Angiotensin-converting-enzyme-2 (ACE-2) is a cell receptor physiologically involved in the activation of the renin–angiotensin–aldosterone system pathway. ACE-2 regulates the activity of angiotensin II (ANG II), a molecule that represents one of the main regulators of systemic blood pressure and, if deranged, plays a critical role in causing hypertension and inducing inflammation [1]. In normal cells, ANG II is converted and neutralized by ACE-2, thus regulating its action and reducing its harmful effects [2]. ACE-2 is constitutionally expressed by epithelial cells of the lungs, heart, kidneys, and blood vessels, on which it provides protection from ANG II [3]. In particular, ACE-2 has been found to be highly expressed in pneumocytes in the lower parts of the human respiratory tract [4]. Further evidence demonstrated the expression of ACE-2 in cardiovascular, digestive, urinary, and reproductive systems [5, 6], spermatogonia, as well as in Leydig and Sertoli cells, ovary, uterus, vagina, and placenta [7], endothelial cells from arterial, and venous vessels [4] and very recently in thyroid cells [8, 9]. The expression of this receptor recently raised great interest in view of the current severe acute respiratory syndrome coronavirus 2 (SARS-CoV-2) pandemic. Indeed, early steps of SARS-CoV-2 infection require the attachment of the *virus* through its S-glycoprotein to the ACE-2 receptor expressed on the host epithelial cells [10].

Identifying the cell subsets expressing ACE-2, which would be susceptible to SARS-CoV-2 infection, and also the factors involved in the regulation of ACE-2 expression by human cells is critical for understanding the pathogenesis of the SARS-CoV-2 and hopefully for developing strategies aimed at increasing the host defence mechanisms. ACE-2 mRNA levels seem to be up-regulated by several factors, including age [11], tobacco smoke [12], and several respiratory infections (e.g., rhinovirus and influenza virus) [13]. Interestingly, the regulation of ACE-2 expression may also be mediated by the canonically antiviral type I and II interferon (IFN) pathways [13, 14]. IFNs are a family of cytokines with a central role in *virus*-directed innate immunity, known for their ability to activate the antiviral state in virus-infected, non-infected, and bystander cells by inducing a gene transcription programme, which, orchestrating various responses, causes interference with different stages of the viral replication cycle [15].

However, SARS-CoV-2 (as well as other coronaviruses) is known to escape from IFN antiviral effects by either indirectly interfering with RNA synthesis [16–18], or directly affecting transcription factors and intracellular signalling molecules that regulate the IFN cascade [19, 20].

Interestingly, some studies reported that ACE-2 expression was predominantly induced by IFNs in various types of cells [12, 21–27] suggesting that IFN therapy or natural coinfections could aggravate COVID-19 by upregulating SARS-CoV-2 entry receptor. However, the fact that IFNs would induce ACE-2 expression was not confirmed by all studies [28, 29]. The expression of ACE-2 was recently found on thyroid cells in primary cultures [8]. The aim of the present study was to investigate whether IFN-γ and tumour necrosis factor alpha (TNF-α) alone or in combination, promote an increased expression of the ACE-2 mRNA in human thyroid cells in primary cultures.

## Materials and methods

### Primary cultures of human thyroid cells

Surgical specimens of normal human thyroid (NHT) were obtained from the contralateral disease-free lobe of patients who underwent thyroidectomy for a solitary benign nodule (*n* = 6). Surgical specimens were minced and then incubated with collagenase type II (Sigma, Saint Louis, MO, USA) 5 mg/ml, in 5 ml of Coon’s F12 medium, for 4 h at 37 °C. Then, 10 ml of Coon’s F12 medium were added, following which, cells were filtered, spun at 1000 × *g* for 10 min, washed with Coon’s F12 medium, spun again, and finally re-suspended in complete medium containing 5% newborn calf serum and a mixture of six hormones including insulin (5 μg/ml), hydrocortisone (50 μg/ml), transferrin (5 μg/ml), somatostatin (10 ng/ml), gly-his-lysine (10 ng/ml), and bovine TSH (1 mU/ml).

### Treatment of NHT with IFN-γ and TNF-α

NHT cells were grown in a complete medium until an 80% confluence was reached. Cells were then detached and seeded in six-well flat plates at a density of 1 × 10^5^ cell/well. After 24 h, the growth medium was removed and cells were incubated for 24 h in serum-free medium containing pro-inflammatory cytokines. Thyroid cells were stimulated with 1000 U/ml IFN-γ (R&D systems, Minneapolis, MN) and with 10 ng/ml TNF-α (R&D Systems, Minneapolis, MN), alone or in combination, for 24 h. These concentrations were chosen on the basis of previous experiments [30, 31]. After 24 h incubation, supernatants were removed and used for CXCL10 assays. Total RNA was extracted and purified from cells treated with the described stimuli.

### Real-time PCR

Total RNA was isolated from thyroid cells treated with IFN-γ and TNF-α, alone or in combination using a total RNA purification kit according to the manufacturer’s instructions (Norgen Biotek, Canada). Genomic DNA was digested using the DNAse enzyme (Norgen Biotek, Canada) at room temperature for 15 min and following the manufacturer’s protocol. Total RNA from samples was reverse transcribed into cDNA using a Sensi Fast c-DNA synthesis kit (Bioline, London, UK), following the manufacturer’s instructions. Real-time PCR was performed using Sensi-Fast SYBR Green Hi-ROX kit (Bioline, London, UK) on a StepOne Plus Applied Biosystems real-time PCR system. Amplification was done under the following conditions: 95 °C for 2 min; followed by 40 cycles of 95 °C, 5 s and 60 °C, 10 s. β-actin and GAPDH were used as endogenous controls. Pre-designed primers targeting human ACE-2 (F: GGGATCAGAGATCGGAAGAAGAAA; R: AGGAGGTCTGAACATCATCAGTG) GAPDH (F: AAATCCCATCACCATCTTCC; R: GGTTCACACCCATGACGAAC) were obtained from Biomers.net GMBH (Soflinger, Germany). Primers of ACE-2 were chosen based on our previous study [8]). All samples were run in triplicate. The expression of ACE-2 in relation to GAPDH was calculated for all the samples.

### ELISA for CXCL10

In order to assess if all primary cultures were responsive to treatment with IFN-γ and IFN-γ + TNF-α, the levels of CXCL10 (a prototype interferon-α-inducible chemokine) [32, 33] were measured in the supernatants. CXCL10 was measured in cell supernatants of NHT cells in NT conditions and after treatment with IFN-γ (1000 U/ml) and TNF-α (10 ng/ml) alone and in combination, using commercially available kits (R&D Systems, Minneapolis, MN). The mean minimum detectable concentration of CXCL10 was 1.67 pg/ml. The intra-assay and inter-assay coefficients of variation were 3.0% and 6.9%, respectively.

### Statistical analysis

Statistical analysis was performed by SPSS (SPSS, Inc., Evanston, IL). Kruskall–Wallis test was used to compare variables with a non-parametric distribution as assessed through positivity to Levene test of equality of variances. Post-hoc analysis was then performed by the Dunn–Bonferroni’s correction for multiple comparisons. Values are reported as mean ± standard deviation (SD) unless otherwise noted. A *p*-value < 0.05 was considered statistically significant.

## Results

### The expression of ACE-2 mRNA by NHT cells is modulated by pro-inflammatory cytokines showing two different patterns of expression

**Fig. 1:**
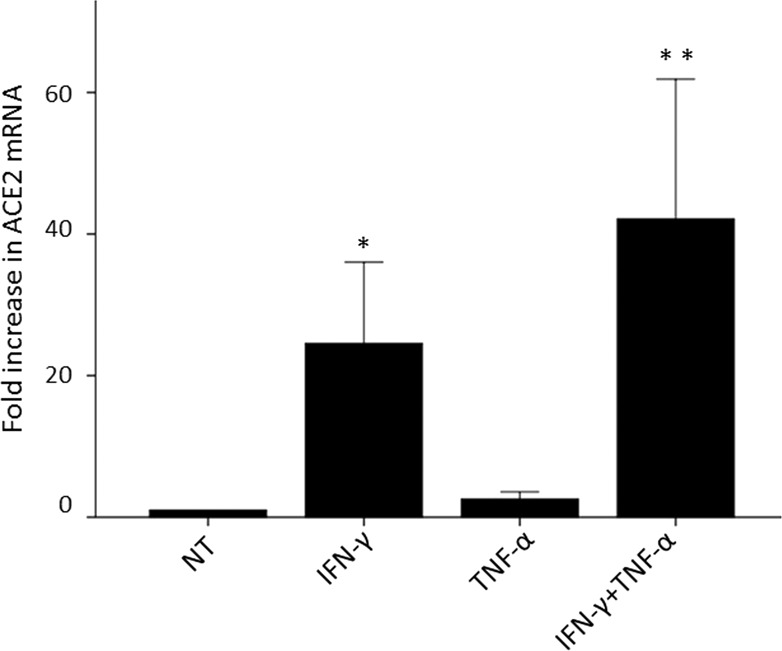
Mean expression of ACE-2 mRNA after treatment with cytokines alone or in combination. Modulation of expression of ACE-2 mRNA after treatment with 1000 U/ml IFN-γ and 10 ng/ml TNF-α alone or in combination in primary cultures of thyroid cells (*n* = 6 samples) after 24 h. The ACE-2 expression levels were normalized on GAPDH and are shown as a change in fold expression compared to not-treated cells. Bars are representative of the mean fold increase in ACE 2 mRNA expression. IFN-γ alone significantly increases the expression of ACE-2 mRNA when compared with not-treated cells (Kruskall–Wallis 63.143, *p* < 0.001; * IFN-γ vs. not-treated *p* < 0.05). Although TNF-α alone does not produce a significant increase in the expression of ACE-2, it strongly synergized with IFN-γ in inducing ACE-2 expression (** IFN-γ + TNF-α vs. IFN-γ *p* < 0.05)

In not-treated (NT) cells, a significant expression of the ACE-2 mRNA was detected in all NHT cultures. As shown in Fig. 1, IFN-γ alone significantly increased the expression of ACE-2 mRNA when compared with NT cells (Kruskall–Wallis 63.143, *p* < 0.001; IFN-γ vs. NT *p* < 0.05). Although TNF-α alone did not produce a significant increase in the expression of ACE-2, it strongly synergized with IFN-γ in inducing ACE-2 expression (IFN-γ + TNF-α vs. IFN-γ *p* < 0.05). However, when the results obtained were analysed for each single cell preparation relevant differences appeared.

**Fig. 2:**
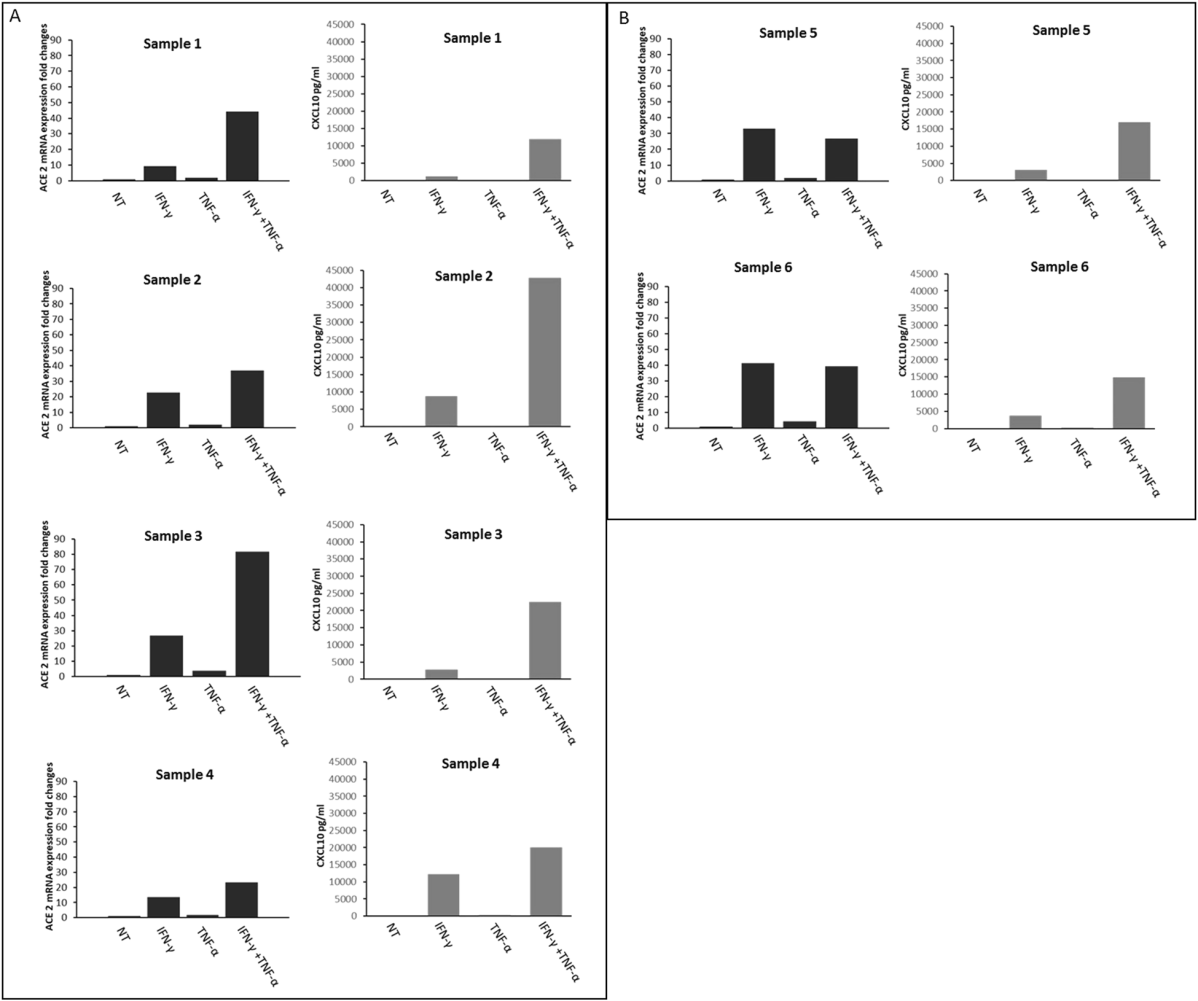
Expression of ACE-2 mRNA and CXCL10 secretion after treatment with cytokines alone or in combination in NHT cells (*n* = 6 samples). (Panel **A**) Modulation of expression of ACE-2 mRNA (on the left) and the secretion of CXCL10 protein (on the right) after treatment with 1000 U/ml IFN-γ and 10 ng/ml TNF-α alone or in combination in primary cultures of thyroid cells (*n* = 4 samples) after 24 h. The ACE-2 expression levels were normalized on GAPDH and are shown as a change in expression fold compared to expression levels in not-treated cells. The CXCL10 secretion levels are shown as protein concentration in each sample. This panel includes all the samples in which the combination of IFN-γ + TNF-α induced a similar response pattern for both ACE 2 and CXCL10 (Cluster 1). In four patients out of six (66.6%), the combination of IFN-γ + TNF-α induces a further increase in ACE-2 expression when compared with the treatment with IFN-γ alone. (Panel **B**) Modulation of the expression of ACE-2 mRNA (on the left) and the secretion of CXCL10 (on the right) after treatment with 1000 U/ml IFN-γ and 10 ng/ml TNF-α alone or in combination in primary cultures of thyroid cells (*n* = 2 samples) after 24 h. The ACE-2 expression levels were normalized on GAPDH and are shown as a change in expression fold compared to expression levels in not-treated cells. The CXCL10 secretion levels are shown as protein concentration in each sample. This panel includes the samples in which the combination of IFN-γ + TNF-α produced a synergic effect in terms of increasing the CXCL10 secretion, but no synergism could be detected for ACE-2 expression (Cluster 2). In 2 patients (33.3%) the expression of ACE-2 after treatment with IFN-γ + TNF-α remained similar to what was observed after treatment with IFN-γ alone

Indeed, as shown in Fig. 2A while in four patients out of six (66.6%), the combination of IFN-γ + TNF-α induced a further increase in ACE-2 expression when compared with the treatment with IFN-γ alone, in the remaining 2 patients (Panel B) (33.3%) the expression of ACE-2 after treatment with IFN-γ + TNF-α remained similar to that observed after treatment with IFN-γ alone. To assess if these results were due to a lack of response to cytokines of these thyroid cultures, the levels of CXCL10 (a prototype interferon-α-inducible chemokine) were assayed in cell culture supernatants of all tested thyroid cultures samples. From this control experiment, we would expect the common response of thyrocytes to the treatment with these cytokines that is: (i) levels of CXCL10 in NT samples will be very low; (ii) treatment with IFN-γ will increase the NT (very low) levels of CXCL10; (iii) treatment with TNF-α alone would not increase NT levels of CXCL10; (iv) IFN-γ + TNF-α would further increase CXCL10 levels induced by treatment with IFN-γ alone. The results obtained showed that all thyroid primary cultures were responsive to the treatment with cytokines as expected. However, it was interesting to observe that the stimulation with the two different pro-inflammatory cytokines alone or in combination yielded a very similar pattern of induction of both ACE-2 mRNA (panel A) and CXCL10 protein in four patients out of six, while in the remaining two patients (Panel B) (33.3%) no such a parallelism was observed.

In detail, in these two latter patients, the combination of IFN-γ + TNF-α did produce a synergic effect in terms of increasing CXCL10 secretion. On the contrary, no synergism was observed when ACE-2 expression was evaluated since similar levels of expression of the ACE-2 mRNA were observed between samples treated with IFN-γ alone and those treated with the combination of IFN-γ + TNF-α.

**Fig. 3:**
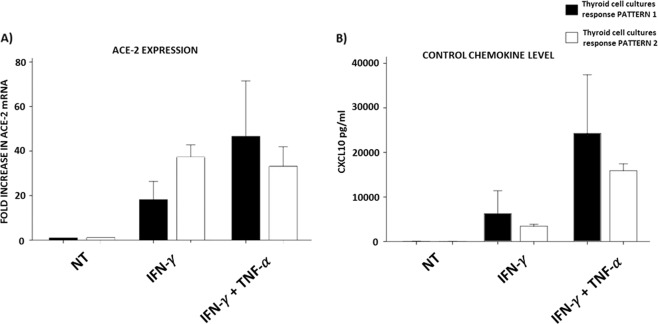
Thyroid cultures samples patterns ACE 2 expression and CXCL10 secretion levels. (Panel **A**) Different ACE-2 expression between Thyroid samples pattern 1 (black) and Thyroid samples pattern 2 (white). (Panel **B**) Similar CXCL10 secretion between Thyroid samples pattern 1 (black) and Thyroid samples pattern 2 (white). Bars are representative of mean fold increase in ACE-2 mRNA

To better clarify this issue, Fig. 3 shows both ACE-2 expression (Panel A) and CXCL10 secretion (Panel B) induced by IFN-γ alone or by IFN-γ + TNF-α. The analysis was performed between samples grouped in two different thyroid sample response patterns. In this view, thyroid sample response pattern 1 represents those samples (4 out of 6) in which an increase of ACE-2 expression was found also after treatment with IFN-γ + TNF-α, whether thyroid sample response pattern 2 represent those samples (2 out of 6) in which an increase of ACE-2 expression was not found after treatment with IFN-γ + TNF-α. Comparing these two different thyroid sample response patterns it emerges that a different response in terms of ACE-2 expression could not be explained by lack of cellular response to TNF-α, because an overlapping effect in terms of CXCL10 secretion was evident among the two patterns (Fig. 3A, B). Thus, when the response of ACE-2 to IFN-γ and to the combination of IFN-γ + TNF-α is examined, it appears that this response is different between thyroid sample response pattern 1 and thyroid sample response pattern 2. This finding would suggest that other factors would be involved in the induction of ACE-2 expression by these two pro-inflammatory cytokines. Although, no conclusive remarks can be drawn, it is interesting noting that thyroid sample response pattern 2 (Panel A), which is the one not responsive to the combination of IFN-γ + TNF-α, is also that in which IFN-γ exert a higher response in terms of ACE-2 increase of mRNA expression.

## Discussion

The results of the present study demonstrate that IFN-γ significantly upregulates the expression of ACE-2 on NHT cells. Previous studies showed that, in different clinical settings, IFN-γ is able to induce ACE-2 expression in human cells [25–27, 34]. This is the first study demonstrating an up-regulation of ACE-2 expression induced by IFN-γ also in human normal thyroid cells.

IFN-γ plays a pivotal role in COVID-19 pathogenesis, being produced by lung resident, dendritic cells and from T helper 1 (Th-1) oriented T-cells recruited at the site of infection, where it inhibits lung epithelial repair after viral recognition [35–37]. Huang et al. found that serum IFN-γ levels were higher in patients with COVID-19 than in healthy individuals as a result of the activation of Th1 cells [38]. Similarly, elevated serum IFN-γ levels were previously reported in patients with severe acute respiratory syndrome coronavirus 1 (SARS-CoV) or Middle East respiratory syndrome (MERS) [39, 40] being also associated with greater viral load and more severe lung damage in COVID-19 [41]. This would imply that increased transcription of ACE-2 by any agent would likely increase viral entry and amplify viral replication.

Moreover, the here reported results show that the expression of ACE-2 was increased also by TNF-α, even if at lower levels as compared to IFN-γ. TNF-α is a pyrogen cytokine released from immune cells in the acute phase of inflammation and infection. It is a central cytokine in viral diseases and is associated with a number of chronic inflammatory diseases [42].

Thus, both IFN-γ and TNF-α are pro-inflammatory cytokines highly upregulated in patients with COVID-19. Interestingly, the recent study by Karki et al. showed that the synergism of these two cytokines is crucial for the induction of inflammatory cell death, inflammation, tissue and organ damage, and mortality of patients infected by SARS-CoV-2 [43]. The synergism between IFN-γ and TNF-α was previously demonstrated in several pathologic conditions. In autoimmune thyroid disease, IFN-γ and TNF-α were consistently reported to synergize in the induction of the chemokine CXCL10 secretion by thyrocytes. The mechanism of this synergism was identified in the fact that TNF-α would up-regulate the membrane expression of IFN-γ receptors [44, 45]. CXCL10 plays a central role in thyroid autoimmune diseases, being involved, in the amplification of the Th-1-oriented immune response and ultimately in the recruitment of infiltrating lymphocytes expressing its receptor CXCR3. This specific subset of T cells constitutively secretes IFN-γ, thus perpetuating the inflammatory process. On the other hand, CXCL10 is also considered a crucial chemokine in COVID-19. Indeed, CXCL10 is among the most studied chemokine in SARS-COV-2 infection, being consistently identified by recent studies as the chemokine playing a crucial role in the SARS-CoV-2-induced cytokine storm [37, 46, 47]. Furthermore, several clinical studies reported that higher CXCL10 circulating levels are positively related with disease severity and increased risk of death in patients with COVID-19.

Based on the notion that elevated levels of CXCL10 are found in both autoimmune thyroid disease and COVID-19, and considering that the synergism between TNF-α and IFN-γ would: (i) promote a more severe course of COVID-19; (ii) strongly increase the secretion of CXCL10 by thyroid cells, the further subsequent hypothesis to be tested was whether a synergic effect could be exerted by IFN-γ and TNF-α also in the induction of ACE-2 expression by thyroid cells.

Our results showed that, as expected, the synergism of IFN-γ and TNF-α consistently produced a strong increase in terms of CXCL10 secretion in all thyroid cultures, while different responses were recorded in terms of ACE-2 expression. Overall two different patterns of response to the combined stimulation with IFN-γ and TNF-α on thyroid cells were registered. The first pattern (thyroid samples response pattern 1) was characterized by a similar behaviour of CXCL10 and ACE-2 in response to the combined stimulation whereas in the second pattern (thyroid samples response pattern 2) the combined stimulation with IFN-γ and TNF-α exerted its expected synergic effects on CXCL10 secretion, while there was no further increase in ACE-2 expression after treatment with the combination of IFN-γ and TNF-α when compared with IFN-γ alone.

The present study was specifically designed to address if IFN-γ and TNF-α alone or in combination would increase the mRNA of ACE-2 in thyroid cells and no mechanistic evaluation was performed in order to explain potential divergences. However, the here reported results might allow the following considerations:

1. The finding that the combined stimulation with IFN-γ + TNF-α synergistically increased CXCL10 secretion in all cell preparations contrasted with the not-univocal synergic effect exerted in terms of ACE-2 mRNA.
2. The lack of synergism of IFN-γ + TNF-α in some samples could be due to a “saturation” of ACE-2 expression obtained by IFN-γ alone. Indeed in these samples, IFN-γ exerted the greatest induction of ACE-2 mRNA, which was not further increased by the combination with TNF-α. These findings would highlight a potential difference among subjects in terms of the mechanisms of induction of ACE-2 expression, which could, at least in part, account for the different degree of severity of SARS-COV-2 infection. This hypothesis deserves to be confirmed by further specific studies.

These results would suggest that elevated levels of pro-inflammatory cytokines could facilitate the entering of the virus in cells by further increasing ACE-2 expression.

## Data Availability

Some or all data used during the study are available from the corresponding author by request.
